# Supplementary material for: Prevalence, patterns and determinants of peripheral neuropathy among leprosy patients in Northeast Ethiopia: A retrospective study
Source: PLoS Negl Trop Dis. 2025 Mar 31;19(3):e0012944. doi: 10.1371/journal.pntd.0012944 (PMC11978083; doi:10.1371/journal.pntd.0012944)
Supplement: Attachment — Submitted filename: Response Letter.docx [file pntd.0012944.s002.docx]

**Response letter**

Dear Editors,

We are grateful for the reviewers' insightful comments. On behalf of my co-authors, we appreciate you and the reviewers for your precious time in reviewing our paper and providing valuable comments on our manuscript (PNTD-D-24-00127).

Your reviews are most helpful. We have carefully reviewed the comments and thoroughly revised the manuscript accordingly. Our responses are given in a point-by-point manner below. We have submitted a revised version of our manuscript. All the changes are marked with track changes.

We are looking forward to your reply on revision. We are very glad to make any further changes that will improve the paper more. Thank you again for your attention and consideration.

Best regards,

Endris Seid, MSc.

**Response to reviewers**

**Reviewer #1:** Are the objectives of the study clearly articulated with a clear testable hypothesis stated? No, please see my detailed comments.

**Response:** Thank you for your valuable feedback on our manuscript titled " Prevalence, patterns and determinants of peripheral neuropathy among leprosy patients at Boru Meda General Hospital, Dessie, Ethiopia: A retrospective study." We would like to appreciate your insightful comments.

Regarding your concern about the clarity of our study objectives and the formulation of a testable hypothesis, we acknowledge that clearer articulation is essential. We aim to address this by refining our objectives as follows:

**Objective 1:** To assess the prevalence of peripheral neuropathy among patients with leprosy.

**Objective 2:** To determine the patterns of leprosy neuropathy (both sensory and motor).

**Objective 3:** To identify the determinants associated with leprosy peripheral neuropathy, including sociodemographic and clinical factors.

In addition to clarifying our objectives, we formulated a hypothesis to guide our analysis. Based on our data and prior literature, our working hypothesis was:

‘‘ In individuals diagnosed with leprosy, the prevalence and severity of peripheral neuropathy are influenced by various factors including age, sex, education level, occupation, residence (urban vs rural) , contact history, duration of the disease, level of skin lesions, symptom onset, WHO classification of leprosy, occurrence of leprae reaction, and family history of leprosy.’’

We believe these revisions will strengthen the manuscript and provide a clearer framework for our study. We look forward to your further feedback and suggestions.

Thank you once again for your time and consideration.

**Reviewer #1:** Is the population clearly described and appropriate for the hypothesis being tested? Partially, please see my detailed comments.

**Response:** Thank you for your comments on our study regarding the prevalence, patterns, and determinants of leprosy peripheral neuropathy. We appreciate your insightful feedback regarding the population description and its relevance to the hypotheses being tested.

We understand the importance of clearly defining the study population to ensure the appropriateness of the hypotheses being tested. In response to your feedback, we have revised the manuscript to provide additional details regarding the inclusion criteria and characteristics of the study population. We have also included a rationale for why this population is relevant to our research questions.

Regarding the population description, our study focuses on patients diagnosed with leprosy who also exhibit symptoms of peripheral neuropathy. We have included all leprosy patients except those who do not fulfill the inclusion criteria. Patients visit the study area(Borumeda hospital) from different parts of Ethiopia, particularly from rural areas where infrastructures are scarce. This population was chosen because leprosy patients are particularly susceptible to peripheral neuropathy in rural areas due to delayed diagnosis and treatment, poor living conditions, limited acess to health care services, nutrition deficiencies and stigma. [World Health Organization (WHO). (2020). Global leprosy (Hansen disease) update, 2019: Time to step-up prevention initiatives. Available online: WHO Global Leprosy Update 2019].

We hope these revisions adequately address your concerns. Please do not hesitate to contact us if you have additional suggestions.

**Reviewer #1:** Is the sample size sufficient to ensure adequate power to address the hypothesis being tested? Yes

**Response:** Thank you for your feedback. Your insights are invaluable to us as we work to ensure the rigor and validity of our findings.

**Reviewer #1:** Were correct statistical analysis used to support conclusions? I think it is ok.

**Response:** We are grateful for your positive feedback.

**Reviewer #1:** Are there concerns about ethical or regulatory requirements being met? Yes.

**Response:** Your positive feedback about ethical or regulatory requirements means a lot to us. It reinforces that our approach is resonating effectively.

**Reviewer #2:** It is not clear whether diagnosis of leprosy patients was done by a trained person. In absence of it, the data can be misleading.

**Response:** Thank you for your valuable feedback on our manuscript titled ‘‘ Prevalence, patterns and determinants of peripheral neuropathy among leprosy patients at Boru Meda General Hospital, Dessie, Ethiopia: A retrospective study.’’ We appreciate your thoughtful consideration of our work.

Regarding your concern about the diagnosis of leprosy patients in our study, we would like to clarify that all diagnoses are done by trained healthcare professionals, dermatologists who have been working for several years on leprosy in the hospital. Some of them even have publications on leprosy.

We acknowledge the importance of ensuring that the diagnostic process is conducted by qualified personnel to maintain the accuracy and reliability of the data. Furthermore, we have included additional details in the methods section of our revised manuscript to explicitly state the qualifications of the personnel involved in diagnosing leprosy cases. This clarification aims to provide transparency and enhance the credibility of our findings.

**Reviewer #1:** Does the analysis presented match the analysis plan? I think it its ok.

- Are the results clearly and completely presented? Partially, I missed the grade of disability at diagnosis.

**Response:** We appreciate the opportunity to address your concerns. Regarding the grade of disability at diagnosis, we acknowledge that this is an important aspect in the context of leprosy management and outcomes. However, it is important to clarify that the primary focus of our study was on the prevalence, patterns, and determinants of leprosy peripheral neuropathy rather than disability assessment at the time of diagnosis.

Our study aimed to investigate specific aspects related to peripheral neuropathy in leprosy, including its prevalence across different demographic and clinical factors, as well as the underlying determinants contributing to its manifestation. These aspects were chosen based on their relevance to understanding the neuropathic complications of leprosy, which remain significant despite advances in treatment and management.

We agree that disability grading at diagnosis could provide valuable additional information. However, due to the scope and specific objectives of our study, we did not include disability grading as a parameter. We believe that focusing on peripheral neuropathy helps to fill an important gap in the literature by shedding light on a critical aspect of leprosy-related morbidity.

In Ethiopia, the	leprosy	disability	management is not well organized and unfortunately almost all hospitals in the country lack a dedicated disability prevention unit. [Federal Ministry of Health, Ethiopia (2017) Final leprosy elimination strategy for Ethiopia from 2016-2020]

Thank you for your time and valuable input.

- Are the figures (Tables, Images) of sufficient quality for clarity? Some data are duplicated in the text and table and figures. It is not necessary.

**Response:** Thank you for your careful review of our manuscript. We appreciate your feedback regarding the duplication of data across the text, tables, and figures. We have carefully examined the manuscript and acknowledge the redundancy identified.

**Reviewer #2:**

1. It is not clear whether these patients are newly diagnosed leprosy patients or patients already on Multi-Drug Therapy.

**Response:** Thank you for your thoughtful review of our manuscript on leprosy peripheral neuropathy. We appreciate your query regarding the status of the patients included in our study with respect to Multi-Drug Therapy (MDT) for leprosy.

In our study, we included both newly diagnosed leprosy patients and those already receiving Multi-Drug Therapy. This diverse inclusion criterion allowed us to examine the spectrum of peripheral neuropathy outcomes in both groups. This clarification will provide transparency regarding the patient population under study and ensure that our findings are appropriately contextualized.

2. In Table 2, all variables are given except disability status at the time of diagnosis, grading of sensory and motor impairment, and whether skin smear was done in a proportion of cases. If this was/is primarily a leprosy centre, then, it should have facilities for skin smear and nerve function assessment.

**Response:** Thank you for your insightful comments and suggestions. We acknowledge the importance of grading sensory and motor impairments in leprosy neuropathy. As you correctly noted, a skin smear test was performed in a proportion of cases as part of routine diagnostic procedures. The study was conducted at a leprosy center with comprehensive diagnostic facilities, including nerve function assessments and skin smear tests, we agree that these details are crucial for understanding our findings within the broader clinical context. We have revised the manuscript to include a dedicated section acknowledging these limitations and their potential impacts on our findings. We appreciate your guidance in helping us improve the clarity and quality of our work.

3. If this data is taken at the time of diagnosis and if all patients are new patients (never treated for leprosy before), then 69.2% of patients presenting with any type of lepra reaction (type 1 or 2) at the time of diagnosis is a very high proportion and requires cross-checking. In the method, two persons have checked the records independently but it is not clear who made the diagnosis (a medical doctor or a nurse) and whether they were properly trained.

**Response:** Thank you for your thoughtful review and valuable feedback on our manuscript on leprosy peripheral neuropathy. We appreciate your query regarding the diagnostic process and the qualifications of those involved.

In our study, all diagnoses of leprosy were made by two experienced dermatologists: a tropical dermatology professional and a health officer with dermatology and leprosy training.

4. As per WHO classification, single or more than one nerve involvement due to leprosy is classified as MB. From Tables 2 and 3, it is not clear whether these patients were included in the MB classification.

**Response:** Thank you for your insightful comments and careful review of our manuscript on leprosy peripheral neuropathy. We appreciate your query regarding the classification of nerve involvement and its inclusion in the MB (Multibacillary) classification as per WHO guidelines. We recognize the importance of adhering to standardized classification criteria for leprosy cases, and we have ensured that our study protocol followed these guidelines meticulously.

We appreciate your diligence in highlighting this aspect, and we will make the necessary revisions to clarify the classification of nerve involvement in our manuscript.

Leproay cases are diagnosed as pauci or multibacillary leprosy, based on clinical and laboratory features. Patients with one to five leprosy skin lesions and one nerve trunk enlargement are diagnosed as pauci-bacillary whereas multi-bacillary (MB) is diagnosed when patients present with six or more skin lesions, less than six skin lesions which have a	positive	slit	skin	smear	result	and	if	there	is enlargement of more than one nerve. Pauci-bacillary cases are treated with two drugs for 6 months while multibacillary ones are treated with three drugs	for	12	months.[ Federal Ministry of Health, Ethiopia (2017) Guidelines for management of TB, DR-TB and leprosy in Ethiopia. Sixth edition, November	2017. Available: https://www.afro.who.int/publications/national-guidelines-tb-drug-resistant-tb-and-leprosy-ethiopia-sixth-edition. Accessed: 30 November 2019.]

5. Out of 380 patients, 115 presented with 0-1 skin lesion, and, 263 patients presented with lepra reaction. From this data, it looks, like all patients with more than 1 skin lesion presented with lepra reaction or several of single skin lesion presented with lepra reaction ? this phenomenon is not common.

**Response:** We appreciate the opportunity to address your queries regarding the relationship between skin lesions and lepra reaction observed in our study.

Regarding your observation that all patients with more than 1 skin lesion appeared to have lepra reaction, or several patients with a single skin lesion also had lepra reaction, we appreciate your attention to this point. We would like to clarify the following:

**Distribution of skin lesions:** Out of the 380 patients included in our study, 115 presented with 0-1 skin lesion, and 263 patients presented with lepra reaction. We acknowledge that this distribution suggests a significant association between the presence of skin lesions and lepra reaction. It is important to note that while our data show a higher prevalence of lepra reaction among patients with more than one skin lesion, not all patients with multiple skin lesions necessarily developed lepra reaction. Similarly, some patients with fewer skin lesions did present with lepra reaction. The observed phenomenon reflects the clinical complexity and variability in the presentation of leprosy neuropathy.

We understand that this phenomenon may seem uncommon or unexpected. To provide a more comprehensive understanding, we plan to incorporate further subgroup analyses to explore the specific characteristics and risk factors associated with lepra reaction among patients with varying numbers of skin lesions as part of our recommendations.

We appreciate your feedback, which will undoubtedly help us refine our manuscript and enhance the clarity of our conclusions. We remain committed to addressing these points in our revised manuscript and look forward to your continued guidance.

6. Figure 1, the graphical presentation of the pattern of peripheral neuropathy is not clear. It should be presented in a tabular form. As per table 2, 140 patients (36.8%) presented with type 1 lepra reaction. It is an acute neuritis form but from the graph (figure 1) it shows less than 5% of patients in both sexes with neuropathic pain. Also, approximately, 20% of patients (both males and females) are shown as presenting with a motor impairment which is a WHO grade 2 disability. In the same figure 1, 60% of males and 80% of females had no impairment whereas in Table 3 it is shown that more 58% patients presented with a single or multiple nerve involvement. Also, some patients are shown as a motor impairment solely. How is it possible that in leprosy patients presenting with a motor deficit without sensory involvement. Was a motor neuron disease ruled out as differential diagnosis ?

**Response:** Thank you for your insightful comments and questions. You noted that according to Table 2, 140 patients (36.8%) presented with type 1 lepra reaction, which typically manifests as acute neuritis. However, Figure 1 indicates less than 5% of patients in both sexes experienced neuropathic pain. This observation raises a valid point. Our study primarily focuses on neuropathic pain associated with leprosy neuropathy. The discrepancy between the percentage of patients with type 1 lepra reaction and those reporting neuropathic pain could be due to several factors, including varying levels of pain perception, differences in reporting pain symptoms, and the complexity of pain assessment in leprosy patients. We discussed the neuropathic pain as part of sensory impairmrnt. will revise the manuscript to discuss these nuances and ensure clarity on this aspect. We acknowledge the discrepancy between motor impairment and sensory involvement as depicted in our figures and tables. Leprosy neuropathy can indeed present with motor deficits without significant sensory involvement, which may indicate predominant motor nerve damage or specific patterns of nerve involvement.

7. In several places, leprosy perihpheral nerve involvement vis-à-vis leprous peripheral neuropathy is used. It is better that authors use leprosy beacuse leprous gives an impression of peripheral neuropathy due to lepromatous leprosy.

**Response:** Thank you for your insightful feedback regarding the terminology used in our manuscript. We appreciate your attention to detail and agree with your suggestion to standardize our terminology to enhance clarity.

We have revised the manuscript accordingly, replacing all occurrences of "leprous peripheral neuropathy" with "leprosy peripheral neuropathy." This adjustment will ensure that our terminology accurately reflects the broader spectrum of peripheral neuropathy associated with leprosy, rather than implying a specific association with lepromatous leprosy alone.

8. Text needs to be adjusted and ancient history e.g., leprosy is 4000 years old etc, is not warranted in the manuscript. Also, authors should quote the latest epidemiological figures for global and Ethiopian leprosy status from the annual leprosy surveillance updates from the WHO weekly epidemiological record.

**Response:** Thank you for your feedback. As suggested, we have revised the manuscript accordingly.

**Reviewer #1:** Are the conclusions supported by the data presented? Partially

**Response:** Thank you for your valuable feedback on our manuscript titled "Prevalence, Patterns, and Determinants of Peripheral Neuropathy Among Leprosy Patients." We appreciate your comments regarding the presentation of conclusions supported by the data.

Upon reviewing your suggestion, we acknowledge the importance of providing a comprehensive and fully supported conclusion based on our findings. We understood that our current conclusions may have been presented partially. To address this concern, we have added the conclusion section to more explicitly connect each key finding with the supporting data from our study.

Specifically, we have:

1. provided a summary of the prevalence of leprosy peripheral neuropathy (LPN) based on our data
2. clearly outlined the identified determinants associated with LPN, such as male sex, advanced age, presence of leprae reactions, presence of more than four skin lesions, longer duration of the disease, and MB leprosy
3. discussed how these findings compare and contrast with existing literature, citing specific studies where our results align or diverge

- Are the limitations of analysis clearly described? No.

**Response:** Thank you for your feedback on our manuscript titled "Prevalence, Patterns, and Determinants of Peripheral Neuropathy Among Leprosy Patients." We appreciate your comments regarding the clarity of the limitations of our analysis.

Upon reviewing your suggestion, we recognized the importance of clearly delineating the limitations of our study. We acknowledge that the current manuscript could benefit from a more explicit description of the methodological and analytical limitations encountered during our research.

To address this concern, we have enhanced the manuscript by providing a detailed discussion on the following key limitations:

1. **Study scope and generalizability:** Our study was conducted in a specific geographical area and over a defined period, which may restrict the generalizability of our findings to broader populations of leprosy patients.
2. **Diagnostic approach:** The prevalence of peripheral neuropathy was estimated based on clinical diagnosis without the use of additional diagnostic tools, which could potentially impact the accuracy of our prevalence estimates.
3. **Causal inference and study design:** Given the cross-sectional design of our study, we acknowledge the inherent limitations in establishing causal relationships between variables. Furthermore, the absence of nerve conduction testing as a confirmatory diagnostic tool may affect the precision of our neuropathy diagnoses.

By explicitly addressing these limitations, we aimed to provide a more comprehensive understanding of the strengths and weaknesses of our study. We appreciate your valuable feedback, which will undoubtedly strengthen the clarity and robustness of our manuscript.

- Do the authors discuss how these data can be helpful to advance our understanding of the topic under study? No

**Response:** Thank you for your thoughtful feedback and for highlighting the importance of discussing how our study data can advance understanding of leprosy peripheral neuropathy.

Our study contributes to understanding of the topic under study in several significant ways:

1. We have analyzed the prevalence and patterns of leprosy peripheral neuropathy], our study provides valuable insights into the epidemiology of this condition. Understanding the prevalence and distribution of leprosy peripheral neuropathy is crucial for targeting interventions and improving patient outcomes.
2. We have examined determinants or risk factors associated with leprosy peripheral neuropathy. These findings shed light on factors that may influence the development or severity of neuropathy in leprosy patients, offering potential avenues for preventive strategies or tailored treatments.
3. Our findings suggest implications for early detection, management strategies, and potentially improving quality of life for individuals affected by leprosy peripheral neuropathy.
4. Our study may also guide some clues for future research efforts in studying leprosy peripheral neuropathy.

In conclusion, our study aims to enhance understanding of leprosy peripheral neuropathy by providing empirical data on its prevalence, identifying associated determinants, and suggesting clinical implications. These insights are crucial for informing healthcare practices, advancing research agendas, and ultimately improving outcomes for patients affected by this debilitating condition.

- Is public health relevance addressed? Yes. Peripheral neuropathy caused by leprosy is a public health problem. The present study highlighted the importance of not forgetting leprosy in Ethiopia.

**Response:** Thank you so much for your kind words and positive feedback. We are pleased to hear this from you.

**Responses to the specific comments:**

1. The manuscript needs a revision for English language.

**Response:** Thank you for your constructive feedback on our manuscript regarding leprosy peripheral neuropathy. We acknowledge your comment about the manuscript needing revision for English language. We have carefully revised the manuscript to ensure clarity and accuracy in language. If you have any specific suggestions or areas of concern regarding the language, we would greatly appreciate your guidance.

2. The name of the microorganism should be presented in italics, following the writing standard for scientific names of biological species.

**Response:** Thank you for your feedback and suggestion regarding the presentation of the microorganism's name in our manuscript of leprosy peripheral neuropathy. We appreciate your attention to detail and agree that scientific names should be italicized according to standard practice. We ensured that all instances of the microorganism's name are correctly formatted in italics throughout the manuscript.

3. The introduction is too generic; it needs to be more specific and guide the reader towards the main objective of the study. The introduction shouldn't only present well-described characteristics about the pathophysiology and classification of leprosy. I suggest it be rewritten to justify the importance and relevance of the study. For example: Ethiopia has declared the elimination of leprosy as a public health problem, yet new cases continue to be diagnosed annually. How many cases are in children? What percentage of grade 2 physical disability at diagnosis? What are the weaknesses of the leprosy control program in Ethiopia and how could this perpetuate the disease transmission in the country? These questions need to be addressed, either in the introduction or in the discussion.

**Response:** Thank you for your constructive feedback on our manuscript. We appreciate your insightful comments regarding the introduction. We agree that the current version may be too generic and will benefit from a more focused approach that clearly justifies the importance and relevance of our study. Specifically, we have incorporated information about the ongoing challenges in leprosy control in Ethiopia, including the persistence of new cases annually, the prevalence of leprosy in children, and the incidence of grade 2 physical disability at diagnosis. Additionally, we have discussed the weaknesses of the leprosy control program in Ethiopia.

4. I think it's important to describe the study setting, the characteristics of the region, and any access and communication difficulties. Could any cultural aspect explain the results found?

**Response:** thank you for your suggestion. It is very crucial point. We have considered it.

5. The literature review needs to be updated. I suggest using the most recent epidemiological data published by the WHO.

**Response:** Thank you for your feedback and recommendation regarding the literature review in our manuscript on leprosy neuropathy. We acknowledge your suggestion to incorporate the most recent epidemiological data published by the WHO. We have ensured that up-to-date information is used, particularly focusing on the latest WHO reports and epidemiological data pertaining to leprosy.

If you have any specific references or additional sources you would like us to consider, please feel free to share them.

6. North America does not only have leprosy in immigrants. Please, see: Belzer A, Ochoa MT, Adler BL. Autochthonous Leprosy in the United States. N Engl J Med. 2023 Jun 29;388(26):2485-2487. doi: 10.1056/NEJMc2302317. PMID: 37379141.

**Response:** Thank you for your insight. We have looked the suggested citation and made correction.

7. Majority is a subset of a group whose number is greater than half of the entire group. In the manuscript it is used with a different meaning.

**Response:** Thank you for your constructive comment. We have revised the meaning used by the term ‘‘ majority’’in the manuscipt.

8. The authors stated that “more than three-quarters of patients, 291(76.6%) had no contact history with leprosy inhabitants previously”. It's important to highlight that they had no known contact with any person affected by leprosy.

**Response:** Thank you very much for your comment. We have highlighted as ‘‘ had no known contact with any person affected by leprosy.’’

9. Table 3 repeats numbers already presented in the text. This is unnecessary.

**Response:** Thank you for your feedback regarding Table 3 in our manuscript on leprosy neuropathy. We appreciate your observation about the redundancy of numbers already presented in the text being repeated in Table 3. We have revised Table 3 accordingly to avoid duplicating information that is already adequately described in the main text.

10. Out of 228 affected nerves. These are not affected nerves, but affected individuals.

**Response:** Thank you for your careful review of our manuscript on leprosy neuropathy.

We appreciate your clarification regarding the terminology. We have changed the text to accurately reflect that the data pertains to affected individuals rather than affected nerves. Specifically, we will correct the phrase "out of 228 affected nerves" to "out of 228 affected individuals" to accurately convey the data.

11. Leprosy is primarily a neural disease; all patients present some degree of clinical or subclinical neuropathy. This needs to be made clear in the text.

**Response:** Thank you for your insightful comment regarding the nature of leprosy as a neural disease and the presence of clinical or subclinical neuropathy in all patients. We appreciate your suggestion to emphasize this aspect more clearly in the text. In our manuscript, we have indeed highlighted the neural manifestations of leprosy extensively.

12. There is no description of the degree of physical disability. This would be a more concrete data to present.

**Response:** Thank you for your valuable feedback and suggestion regarding the inclusion of the degree of physical disability in our study on the prevalence, patterns, and determinants of leprosy neuropathy. We appreciate your thoughtful consideration of our work.

Regarding the absence of this data, we opted not to include a description of the degree of physical disability in this particular study for the following reasons:

1. **Study Focus:** our primary aim was to investigate the prevalence, patterns, and determinants of leprosy neuropathy among the study population. We aimed to specifically analyze neuropathic complications and associated factors rather than broader measures of disability.
2. **Previous Research:** Existing literature has extensively covered physical disability assessments in leprosy, and our study sought to complement these findings by providing novel insights into neuropathic manifestations and associated determinants.

13. The authors need to inform how the active case-finding system works in the study area. Without contact, there is no transmission of M. leprae.

**Response:** we thank you for your insight, we have revised the manuscript and we highlighted the case finding system.

14. The discussion presents some hypotheses apparently without scientific basis. For example: “The predominance of PB forms in our population may be due to relatively good immunity against leprosy.” I think the most plausible hypothesis in this case is that the patients were misclassified as PB.

**Response:** Thank you for your thoughtful feedback on the discussion section of our manuscript on leprosy neuropathy.

We appreciate your observation regarding the hypotheses presented, especially concerning the predominance of PB forms in our population. We understand your concern about the scientific basis of the hypothesis suggesting good immunity against leprosy. Upon reflection and considering your suggestion, we agree that an alternative hypothesis, such as misclassification of patients as PB, may indeed be more plausible.

We have seen the discussion section to carefully reconsider and reframe our hypotheses based on more grounded scientific reasoning, including the possibility of misclassification affecting the distribution of PB forms in our study population.

15. The authors should cite this important systematic review and meta-analysis about the risk factors for physical disability in patients with leprosy. de Paula HL, de Souza CDF, Silva SR, et al. Risk Factors for Physical Disability in Patients With Leprosy: A Systematic Review and Meta-analysis. JAMA Dermatol. 2019;155(10):1120–1128. doi:10.1001/jamadermatol.2019.1768

**Response:** Thank you for your comment and for bringing the systematic review and meta-analysis by de Paula et al. to our attention. We agreed that this reference is highly relevant to our study on the prevalence, patterns, and determinants of leprosy neuropathy. Thus, we have incorporated the citation of the following article into the revised manuscript:

de Paula HL, de Souza CDF, Silva SR, et al. Risk Factors for Physical Disability in Patients With Leprosy: A Systematic Review and Meta-analysis. JAMA Dermatol. 2019;155(10):1120–1128. doi:10.1001/jamadermatol.2019.1768

16. Replace references 6 and 7 with Weekly epidemiological record 15 SEPTEMBER 2023, 98th YEAR / No 37, 2023, 98, 409–430 chrome-extension://efaidnbmnnnibpcajpcglclefindmkaj/https://cdn.who.int/media/docs/default-source/weekly-epidemiological-record/wer9837-eng-fre.pdf

**Response:** Thank you for your comment and for providing the specific reference from the Weekly Epidemiological Record of September 15, 2023. We appreciate your attention to detail in ensuring the accuracy and relevance of our citations. As sugessted, we have replaced references 6 and 7 in our manuscript with the following source: Weekly epidemiological record 15 SEPTEMBER 2023, 98th YEAR / No 37, 2023, 98, 409–430.

17. Reference 61 = 58, it's repeated.

**Response:** Thank you for your observation regarding the duplication of references in our manuscript on the prevalence, patterns, and determinants of leprosy neuropathy.

We have identified the issue where Reference 61 is a duplicate of Reference 58. We apologize for this oversight.

To address this, we carefully reviewed our reference list and ensured that each citation is unique and correctly corresponds to the appropriate source.

Thank you for bringing this to our attention. Your feedback is instrumental in improving the accuracy and clarity of our work.
