# Supplementary material for: Prevalence, patterns and determinants of peripheral neuropathy among leprosy patients in Northeast Ethiopia: A retrospective study
Source: PLoS Negl Trop Dis. 2025 Mar 31;19(3):e0012944. doi: 10.1371/journal.pntd.0012944 (PMC11978083; doi:10.1371/journal.pntd.0012944)
Supplement: Attachment — Submitted filename: Response_Letter_auresp_2.docx [file pntd.0012944.s003.docx]

**Response letter**

Dear Editors,

On behalf of my co-authors, I’d like to appreciate you and the reviewers for taking your precious time in reviewing and providing valuable comments on our manuscript (PNTD-D-24-00127).

Your reviews are very important. We have carefully reviewed the comments and thoroughly revised the manuscript accordingly. Our responses are given in a point-by-point manner below. We have submitted a revised version of our manuscript. All the changes are marked with track changes.

We are looking forward to your reply on revision. We are very glad to make any further changes that will improve the paper more. Thank you again for your attention and consideration.

Best regards,

Endris Seid, MSc.

**Response to reviewers**

**Reviewer #1:** - Are the objectives of the study clearly articulated with a clear testable hypothesis stated? Yes.

**Response:** Thank you for your valuable feedback on our manuscript titled ‘‘ Prevalence, patterns and determinants of peripheral neuropathy among leprosy patients at Boru Meda General Hospital, Dessie, Ethiopia: A retrospective study.’’ We are thrilled to hear this from you.

**Reviewer #1:** - Is the study design appropriate to address the stated objectives? Yes.

**Response:** Thank you for your affirming feedback. We are delighted to hear that you found our effort useful.

**Reviewer #1:** - Is the population clearly described and appropriate for the hypothesis being tested? Yes.

**Response:** Thank you for your positive response. Your insights are invaluable to us.

**Reviewer #1:** - Is the sample size sufficient to ensure adequate power to address the hypothesis being tested? Yes,
**Response:** Thank you very much for your feedback.

**Reviewer #1:** - Were correct statistical analysis used to support conclusions?
I think it is ok.

**Response:** Thank you for your appreciable feedback regarding the statistical analyses used in our study. We appreciate your acknowledgment that the analyses are acceptable for supporting our conclusions. We would appreciate your feedback on any particular areas you believe need improvement or suggestions you may have for bolstering our analysis in subsequent studies.

We genuinely appreciate your support and insights and Thank you once again for your thoughtful review.

**Reviewer #1: -** Are there concerns about ethical or regulatory requirements being met? I think it is ok.

**Response:** Your positive feedback about ethical or regulatory requirements means a lot to us. It reinforces that our approach is resonating effectively.

**Reviewer #1:** -Does the analysis presented match the analysis plan?

I think it its ok.

**Response:** We thank you for your feedback. We would be very grateful for your advice if you have any particular recommendations for enhancement in this area.We realize that the integrity of our study depends on strictly following the analytical plan, and your advice is really helpful to us in making sure we uphold this quality.
**Reviewer #1:** -Are the results clearly and completely presented?
Partially, I missed the grade of disability at diagnosis. However, it seems the authors do not have this data. So, once they declare it as a study limitation in the discussion, it will be ok for me.

**Response:** Thank you for your thoughtful feedback regarding the clarity and completeness of our results and we appreciate your observation about the absence of data on the grade of disability at diagnosis.

We recognized the importance of this information and agree that its absence is a limitation of our study. As you suggested, we will ensure to clearly acknowledge this limitation in the discussion section.

**Reviewer #1:** -Are the figures (Tables, Images) of sufficient quality for clarity? Ok.

**Response:** Thank you for your constructive response.

**Reviewer #1:** -Are the conclusions supported by the data presented? Yes.

**Response:** Thank you for your insightful feedback.

**Reviewer #1:** -Are the limitations of analysis clearly described? Can be improved. Please see my detailed comments

**Response:** We are grateful for your constructive response.
**Reviewer #1:** -Do the authors discuss how these data can be helpful to advance our understanding of the topic under study? Ok.

**Response:** Thank you for your thoughtful feedback.
**Reviewer #1:** -Is public health relevance addressed? Yes. Peripheral neuropathy caused by leprosy is a public health problem. The present study highlighted the importance of not forgetting leprosy in Ethiopia.

**Response:** Thank you for your encouraging feedback.

**Reviewer #1: Specific comments:**

**1.**  Change “leprae reaction” to “leprosy reaction”.

**Response:** Thank you for your comment of using more precise term. We appreciate your feedback and we have changed.
**2.**   Leprosy is primarily a neural disease; all patients present some degree of clinical or subclinical neuropathy. This needs to be made clear in the text. In the outcomes definitions, leprosy peripheral neuropathy is defined as: “functional impairment and/or structural damage to sensory, motor and autonomic nerve fibres within the peripheral nervous system.”
I suggest the authors should include the following: “detected by clinical nerve palpation (enlarged nerve, pain or feeling of electric shock)” without the use of ultrasound, electroneuromyography, or any complementary examination.

**Response:** We are grateful for your insight. We have considered your suggestion.
**3.**    There is no description of the degree of physical disability. This would be a more concrete data to present. I think it should be mandatory to talk about physical disabilities when the authors intend to investigate the prevalence and patterns of leprosy neuropathy. Once it seems the authors do not have these data, I suggest including this in the discussion as a study limitation.

**Response:** Thank you for your thoughtful feedback and we agree with your feedback and we took it as a limitation.

**Reviewer #2:** -Authors need to adjust the text according to the patients (new) presenting with peripheral neuropathy or with lepra reaction at the time of diagnosis and those who worsened during the course of MDT or after completion of treatment.

**Response:** Thank you for your valuable feedback on our manuscript titled ‘‘ Prevalence, patterns and determinants of peripheral neuropathy among leprosy patients at Boru Meda General Hospital, Dessie, Ethiopia: A retrospective study.’’ We are grateful for the very important comment you rasised. The data used is a secondry data, from patients cards which make it difficult to describe in such a way you sugessted. Looking cards again, we at least tried to put figures on how many of the patients were new cases and on MDT. Out of 380 patients, 120 were new and the remaining 260 were repeat cases. The number of patients with leprosy reaction has already been delineated.

**Reviewer #2:** -Authors should also provide a separate table on peripheral neuropathy, lepra reaction vis-a-vis the type of disease (PB, MB).

**Response:** Thank you for your comment. We made the corection as suggested.

**Reviewer #2:** -Nerve involvement in table 2 (clinical characteristics of patients) is missing. In the same table duration of disease should be qualified with at the time of presentation as a new case.

**Response:** We thank you for your critical review. We made the necessary adjustment as you sugessted.

**Reviewer #2:** - Please specify when you say that this hospital sees 20 leprosy patients per month. Does this mean 20 new cases or it includes cured patients coming back for their nerve damage consultation?

**Response:** Thank you for your insightful comment. It includes all patients. The number mentioned here is the average number of cases (both new and those on follow up) seen each month.

**Reviewer #2:** - It is not clear why authors avoided to follow WHO grading of voluntary muscle testing.

**Response:** Thank for your review. Since we used secondary data, it’s difficult to address every thing. We acknowledge your relevant comment and we take it as a limitation.

**Reviewer #2:** - Authors should add a separate table on sensory and voluntary muscle testing grades for eyes, hands and feet. It is surprising that none of the patient presented with eye involvement.

**Response:** Thank you for your comment dear reviewer. To highlight sensory, and voluntary muscle testing grades for the eyes, hands, and feet on separate table, patient records, we analayzed data from, did not provide specific details about eye involvement or the grading of muscle testing for these areas. We have noted this limitation and we recognized the fact that this is an important aspect that could have improved understanding of our findings. We agree that the absence of eye involvement in our patient population is intriguing and may warrant further investigation in future studies.

**Reviewer #2:** - Since authors have not followed the latest WHO criteria to classify patients as PB and MB, they should mention it in the methods or as a separate note on limitations of the study.

**Response:** Thank you for your feedback. We used WHO - PB/MB classification. We would be glad if we addressed your specific concern.

**Reviewer #2:** - Whether nerve thickening without any sensory, motor or autonomic involvement is taken into consideration.

**Response:** Thank you for your insightful feedback. This is one of the limitation we agreed with.

**Reviewer #2:** - How many patients were of pure neuritic leprosy (no skin involvement)?

**Response:** Thank you for your feedback. In table 2, we presented patients with skin lesions as 0-1 to be 115(30.3%), out of this 40 patients had 0 (no) skin involvement. They are included in skin lesion of 0-1 range.

**Reviewer #2:** -Authors should also provide a separate table on peripheral neuropathy, lepra reaction vis-a-vis the type of disease (PB, MB).

**Response:** Thank you for your feedback. We have provided a separate table on peripheral neuropathy, lepra reaction vis-a-vis the type of disease (PB, MB).

**Reviewer #2:** -On page 8, out of 228 affected individuals, 94 (41.2%) patients' nerve impairments are described as not specified. What does this mean? Either patient is suffering from nerve involvement or not suffering. If this is not specified, then why this number has been taken into calculations.

**Response:** Thank you for your essential feedback. We have revised and put correct figures.

**Reviewer #2:** - In the abstract, authors should mention the prevalence finding of this analysis.

**Response:** Thank you for your feedback. We are grateful for your suggestion.

**Reviewer #2:** - It is not clear why authors are mentioning 10-15 years old leprosy data of the World and Ethiopia and why not giving the latest figures that is readily available publicly in WHO's weekly epidemiological report on global leprosy surveillance. Authors seem to be aware about availability of this data as they have already given this reference but from previous years.

**Response:** Thank you for your feedback. Infact, there are some old figures used but they are very much inline with the study we are working on.

**Reviewer #2:** - In the Introduction section, WHO figures have been given without the reference.

**Response:** Thank you for your feedback. We didnot left figures without reference. We may rather used a paragraph with single reference; more than one sentences may have the same source.

**Reviewer #2:** - WHO target of leprosy elimination of less than 1 case per 10,000 population is defined as a public health problem target and not as elimination of disease (zero case). Therefore, new cases are expected to come for foreseeable future.

**Response:** Thank you for your feedback. We mentioned similar concept.

**Reviewer #2:** - On page 2, last paragraph, please include lateral popliteal nerve as one of the affected nerves.

**Response:** Thank you for your feedback. That is important insight. We have included lateral popliteal nerve as one of the affected nerves.

**Reviewer #2:** - If more than 40 years and above is one of the determinants then how authors have ruled out peripheral neuropathy due to micronutrient deficiencies (both in males and females) and whether history of smoking and tobacco is taken?

**Response:** Thank you for your feedback. We used diagnoses made by experts in leprosy and we just took the dignosis from patient cards.

**Reviewer #2:** - Figure 1 distribution of pattern of leprosy peripheral neuropathy: In leprosy, sensory impairment is the first sensation to go, therefore, motor impairment without sensory impairment should be ruled out for a motor neuron disease. Section on patterns of neuropathy needs more clarification and description. From this graph, and from the tables, it is not clear whether steroids were prescribed in lepra reaction patients and what was the impact of it on the nerve impairments. Generally, with an adequate course, majority patients improve without any residual disabilities especially acute neuritis cases. It is also important to note, how type 2 lepra reaction patients were managed? whether clofazimine were prescribed to them and what was the outcome.

- Proof reading is needed for better clarity on drafting.

- Overall major improvements are needed in the manuscript.

- Several of references are quoted from previous articles from this centre. This should be avoided and original references should be given.

**Response:** Thank you for your indepth feedback. Your comments are very crucial to us. We appreciate the opportunity to address your concerns. We mentioned points that we have not addressed as limitations.

**Reviewer #2: specific comment:** This work and the observations are important for leprosy control in Ethiopia but data should be presented in line with the title of the article.

**Response:** Thank you so much for your kind words and positive feedback. We are pleased to hear this from you.
